# Dental Implants and Orthodontic Mini-Screws in a Patient with Undiagnosed Von Willebrand’s Disease: A Case Report

**DOI:** 10.3390/dj12120381

**Published:** 2024-11-25

**Authors:** Alessandro Bruni, Francesca Giulia Serra, Andrea Abate, Alessandro Ugolini, Cinzia Maspero, Francesca Silvestrini Biavati, Valentina Lanteri

**Affiliations:** 1 Surgical, Medical and Dental Department, University of Modena and Reggio Emilia, 41121 Modena, Italy; alebruni@unimore.it (A.B.); valentina.lanteri@unimore.it (V.L.); 2 Private Practice, 10019 Strambino, Italy; francesca.g.serra@gmail.com; 3 Department of Sciences Integrated Surgical and Diagnostic, University of Genova, 16126 Genoa, Italy; alessandro.ugolini@unige.it (A.U.); silvestrini.fra@gmail.com (F.S.B.); 4 Fondazione IRCCS Cà Granda, Ospedale Maggiore Policlinico, 20122 Milan, Italy; 5 Department of Biomedical Surgical and Dental Sciences, University of Milan, 20122 Milan, Italy

**Keywords:** orthodontic mini-screw, dental implant, prosthetic dentistry, Von Willebrand

## Abstract

**Background:** Dental implants are commonly employed to address edentulism, while orthodontic treatments often incorporate mini-screws to enhance tooth movement and provide stable anchorage. Both procedures are integral to modern dental practice and frequently interact in comprehensive care scenarios. While oral health professionals routinely assess patients’ medical histories before procedures, undiagnosed coagulopathies, such as Von Willebrand Disease (VWD), can present significant challenges when invasive procedures are carried out, such as the insertion of implants or mini-implants. **Case description:** This case report discusses the surgical placement of dental implants and orthodontic mini-screws in a patient with previously undiagnosed VWD, underscoring the potential complications and the importance of recognizing bleeding disorders in clinical practice, and provides some advice on the management of patients with previously undiagnosed VWD after/during surgical procedures. **Conclusions:** To prevent the risk of excessive bleeding, before surgery, all patients should be screened through precise questions on bleeding history.

## 1. Introduction

Von Willebrand Diseases (VWDs) comprise a spectrum of hemorrhagic disorders characterized by either a quantitative or qualitative defect in the Von Willebrand factor (VWF) [1]. The prevalence of VWD in the general population is estimated to range from 0.6% to 1.3%, including all forms of the disorder [2,3]. However, symptomatic VWD requiring specific treatment occurs at a much lower frequency, approximately 1 in 10,000 individuals, with VWD Type 1 accounting for approximately 70–80% of all diagnosed cases of VWD, making it the most common subtype, and with Type 3 VWD being extremely rare, affecting around 1 in 1,000,000 people. However, the symptomatic prevalence in the general population is estimated to be lower, due to its often mild presentation [4]. Despite these statistics, many cases remain undiagnosed for extended periods, leading to undetected bleeding complications in various clinical settings [5].

VWD is typically inherited as an autosomal dominant trait, although autosomal recessive inheritance is observed in rarer forms of the disease [6]. Depending on the type and severity of VWD, the disease commonly involves prolonged bleeding times, factor VIII deficiency, and defective platelet adhesion [6]. VWD Type 1 is the most prevalent and usually presents with mild symptoms, although severe bleeding may occasionally occur [7]. The ABO blood group also plays a role in the manifestation of the disease, with individuals of blood group O exhibiting lower baseline VWF levels compared to other blood groups [7]. Diagnosing VWD requires a comprehensive panel of tests, as no single assay can confirm the functionality of VWF [8]. Screening typically includes evaluating VWF levels, platelet adhesion capabilities, and the interaction between VWF and factor VIII [9].

In the context of dental practice, particularly during extraction procedures, managing hemorrhage in patients with undiagnosed bleeding disorders like VWD can be especially challenging [10]. While acquired coagulopathies are often identified through a detailed medical history, congenital conditions such as VWD frequently remain asymptomatic and undiagnosed until a bleeding event occurs. This presents a significant risk during surgical procedures, as patients may unexpectedly experience excessive intra- or post-operative bleeding. Although reports of severe blood loss due to VWD are more common in other surgical specialties, such cases in dentistry, though less frequent, can still pose considerable risk. Dental implants represent one of the most valued and widely used solutions for the rehabilitation of edentulism. However, their insertion presents challenges in patients with systemic conditions affecting coagulation [11]. Notably, Von Willebrand’s Disease (VWD) introduces significant risks due to impaired blood clotting. Despite the widespread use of implants, the literature on cases of previously undiagnosed VWD patients remains limited [11,12,13,14].

This case report underscores the importance of recognizing undiagnosed bleeding disorders, especially VWD, in dental patients, and emphasizes the need for practitioners to maintain a high level of competence in managing unforeseen hemorrhagic events. The prompt recognition and effective management of such complications are vital to ensuring patient safety in routine dental procedures.

## 2. Case Report

**Figure 1:**
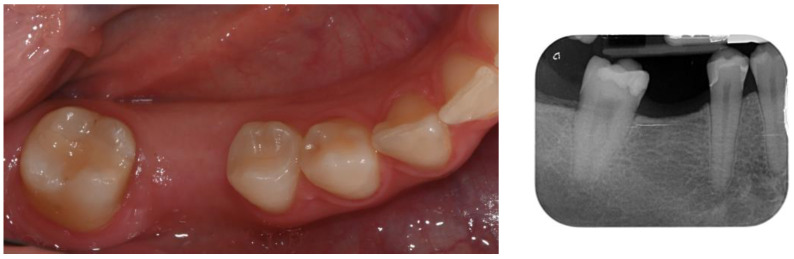
Edentulous zone due to the extraction of the first lower right molar.

In December 2021, a 25-year-old female patient presented to our clinical private practice for comprehensive care and the rehabilitation of single missing tooth (4.6) (Figure 1). Five years before, the patient had undergone a tooth extraction due to caries and had also received other dental treatments, including restorations and the extraction of the tooth 4.8.

The patient reported no significant history of mucocutaneous bleeding, hemarthrosis, or menorrhagia, nor any excessive bleeding following dental extractions or accidental work-related injuries.

**Figure 2:**
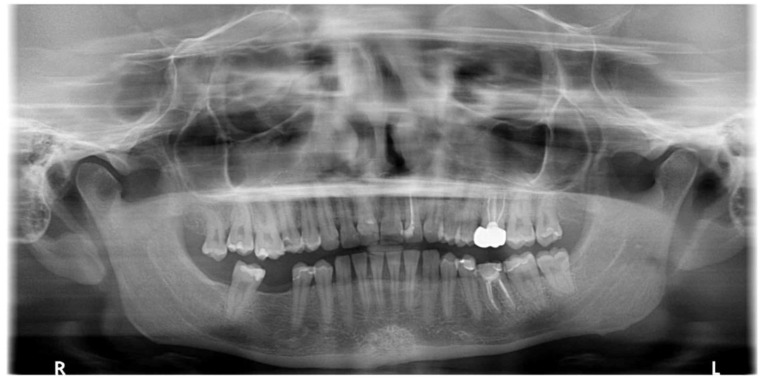
Orthopantomography of the patients before treatment.

**Figure 3:**
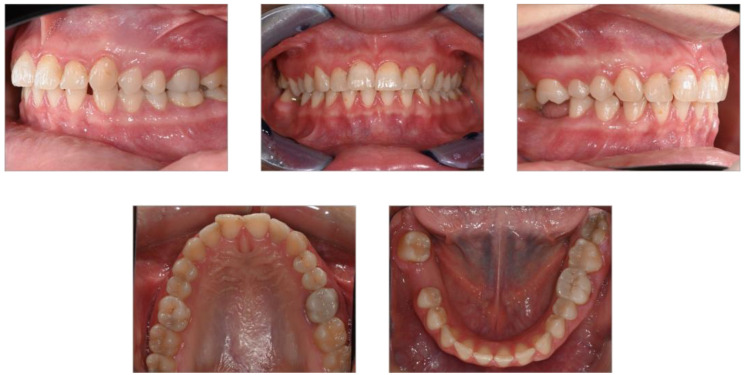
Intra-oral photos of the patient.

Overall, the patient maintained good health, followed a healthy lifestyle, and was a non-smoker. While there was no reported history of trauma, she did mention having experienced hematomas and notable bleeding after a scaling procedure in 2018, which required medical attention. The hematological test results at that time, including PT and PTT, were within the normal limits, leading to the conclusion that no further investigation was deemed necessary. A dental examination revealed multiple caries, previous restorations, and gingivitis associated with plaque and tartar accumulation. During the intraoral examination, the patient exhibited gingivitis associated with calculus and plaque, with a plaque index (PI) of 60% and bleeding on probing (BOP) of 50%. Additionally, teeth 1.8 and 1.6 were found to be extruded, likely due to the absence of teeth 4.8 and 4.6, respectively (Figure 2 and Figure 3).

The treatment plan consisted of professional oral hygiene and scaling to reduce PI and BOP, to be repeated every 4 months until the end of the prosthetic and orthodontic treatment.

The patient underwent a professional dental cleaning session, followed by necessary restorative treatments, without any significant bleeding occurring during the procedures. As a non-smoker, her follow-up examination revealed plaque and bleeding indices below 20%, confirming her suitability for implant-prosthetic rehabilitation in the edentulous site.

**Figure 4:**
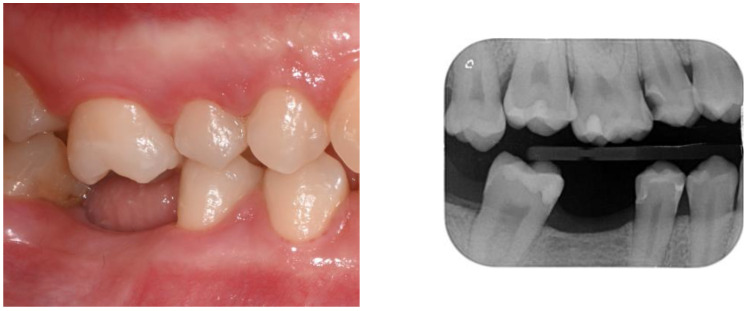
Edentulous zone and the overeruption of tooth 1.6.

For the prosthetic treatment plan, considering the overeruption of tooth 1.6 and the extrusion of 1.8, a decision was made in consultation with the orthodontist to first proceed with the extraction of 1.8, followed by the intrusion of 1.6 to optimize the occlusion (Figure 2 and Figure 4).

Following an initial evaluation, documentation was gathered for a comprehensive orthodontic diagnosis. This included extraoral and intraoral photographs, a prescription for a lateral cephalometric radiograph to perform a cephalometric analysis, and an intraoral scan.

The patient was presented an orthodontic treatment plan that could be carried out using either clear aligners or a fixed multi-bracket therapy. Both options would involve the use of auxiliaries (mini-orthodontic screws) over a duration of 24 months. The treatment would culminate in the placement of fixed and/or removable retainers for an indefinite period. The patient chose not to pursue orthodontic treatment and decided instead to proceed solely with the implant rehabilitation and the pre-prosthetic orthodontic treatment, which involved the intrusion of tooth 1.6.

In agreement with the orthodontist, the extraction of tooth 1.8 was succeeded by the orthodontic intrusion of tooth 1.6. After the extraction of 1.8 in December 2021, the patient did not report any unusual bleeding at the extraction site. However, 10 days later, she noted the development of extensive ecchymosis on her right arm. After contacting her primary care physician, she underwent coagulation screening tests, which yielded the following results:

Thrombocytosis: 238,000/µL.

PT INR: 1.07 (normal value: 0.80–1.20).

APTT: 35.3 s.

APTT ratio: 1.12 (normal value: 0.80–1.18).

Fibrinogen level: 394 mg/dL.

A CBCT scan was performed on the patient to assess the alveolar ridge in preparation for the placement of an implant in the 4.6 region. This allowed for precise evaluation of the bone density and quality, as well as accurate measurement of the distance between the alveolar crest and the mandibular canal, ensuring the optimal positioning of the implant while avoiding impingement of the inferior alveolar nerve [15].

**Figure 5:**
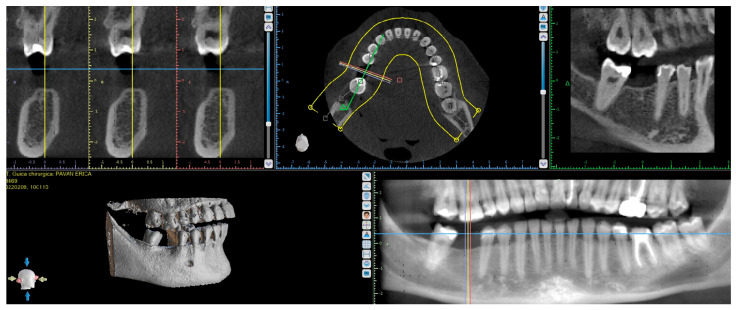
CBCT of patient before implant placement.

The edentulous site in the 4.6 region was classified as Class 3 according to the Cawood and Howell classification [16], indicating moderate bone resorption with a well-rounded ridge. Despite the partial bone loss, the ridge shape remained suitable for implant placement without the need for immediate augmentation procedures. The CBCT scan confirmed that the bone quality and density were adequate, and the ridge’s dimensions were sufficient to facilitate implant placement (Figure 5).

Moreover, it was observed that the resorption of the buccal alveolar wall required gingival augmentation with a connective tissue graft along with the implant placement for the correction of the bone defect.

**Figure 6:**
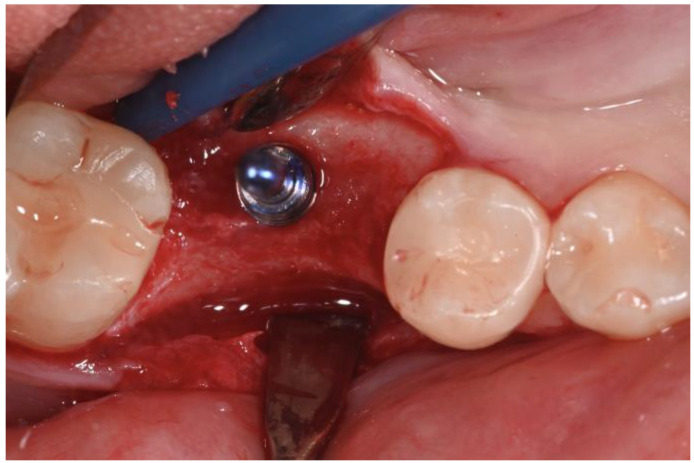
Full-thickness flap elevation and preparation of the implant site.

A two-stage surgical approach was planned: the first stage involved the insertion of the implant screw, followed by a second stage 4 months later for uncovering the implant and simultaneously increasing the connective tissue with a roll flap technique (Figure 6).

Before surgery, verbal and written consent was obtained, and the patient received a prophylactic dose of amoxicillin clavulanate (1 g) to prevent postoperative infections [17].

**Figure 7:**
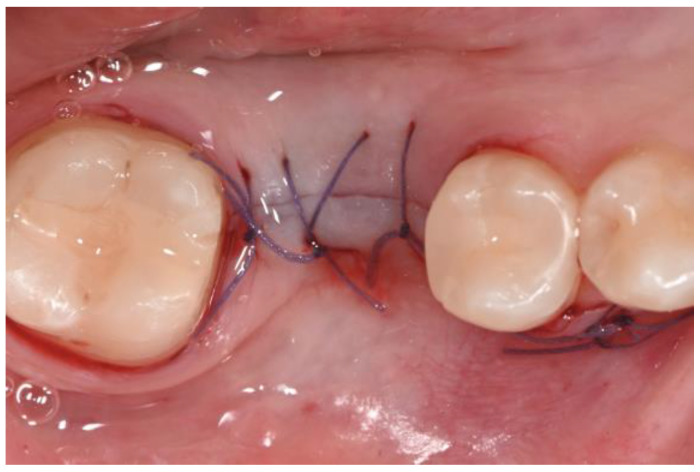
Flap was sutured with resorbable suture.

**Figure 8:**
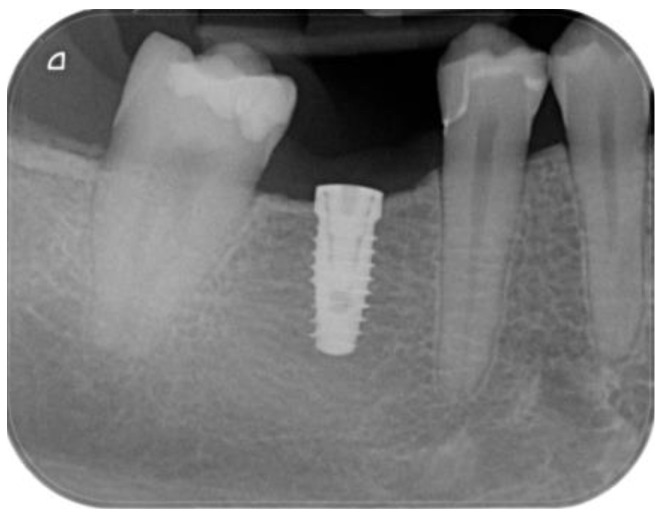
Intra-oral control X-ray.

Local anesthesia was administered using mepivacaine with adrenaline (1:100,000) through infiltration. A crestal incision was made, and a full-thickness mucoperiosteal flap was reflected. After site preparation using dedicated burs, a bone-level implant measuring 4 × 10 mm (BIOMAX, Planegg, Germany) was placed. The flap was sutured using resorbable Vycril 5.0, and an intra-oral control X-ray was performed (Figure 7 and Figure 8).

**Figure 9:**
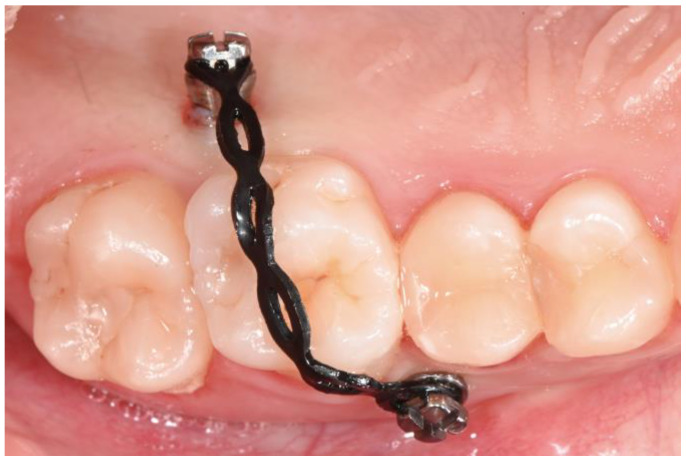
TAD-supported intrusion mechanics.

Then, two orthodontic interradicular mini-screws measuring 8 mm × 1.5 mm (HDC, Vicenza, Italy) were inserted vestibularly between teeth 1.5 and 1.6 and palatally between teeth 1.6 and 1.7 after the infiltration of 1/5 cartridge of 1.70 mL of mepivacaine 2% with 1:100,000 adrenaline. An elastic power-chain was applied at the head of the two mini-screws, passing over the occlusal surface of tooth 1.6 (Figure 9).

To minimize the risk of post-operative complications, the patient was given specific recommendations, including a liquid and cold diet, the application of ice packs for the first few hours, and avoiding hot drinks. Additionally, amoxicillin clavulanate (1 g) and ibuprofen (600 mg) were prescribed, and the patient was instructed to rinse twice daily with chlorhexidine digluconate 0.12.

**Figure 10:**
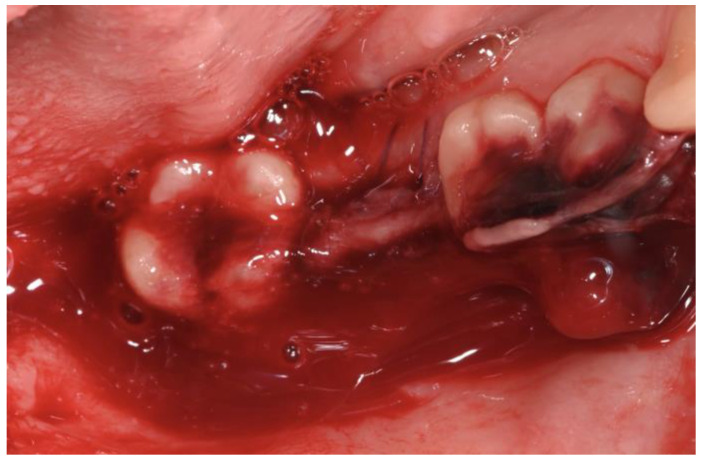
Excessive bleeding after 24 h.

After 24 h, the patient returned reporting excessive bleeding from the implant site. Despite direct pressure with non-resorbable gauze and the local application of tranexamic acid, the bleeding did not stop (Figure 10). The patient was promptly referred to a hematologist, who prescribed oral tranexamic acid (two ampoules of 5 mL, every 8 h).

Consequently, specific hematological parameters were assessed to evaluate coagulation.

The laboratory test results were as follows:

- FVIII = 43% (normal range: 50–200).
- VWF antigen (quantitative) = 35% (normal range: Group O: 48–160; Groups A, B, AB: 50–210).
- VWF R:CoF (qualitative) = 32.5% (normal range: Group O: 44–165; Groups A, B, AB: 54–210).
- PFA-100: CT/EPI (platelet function test) = >300 (normal range: 74–191) CT/ADP (platelet function test) = >300 (normal range: 57–152).

The values of FVIII, VWF antigen, and VWF R:CoF were found to be lower than the normal range for the patient’s blood group, which was O positive. Based on these findings, the hematologist confirmed the diagnosis of vWD type 1. An a posteriori Bleeding Assessment Test (ISTH-BAT) was performed (https://practical-haemostasis.com/Clinical%20Prediction%20Scores/Formulae%20code%20and%20formulae/Formulae/Bleeding-Risk-Assessment-Score/ISTH_BAT_score.html, accessed on 1 October 2024), yielding a score of 6.

The results of a complete blood count, aggregation tests, and a platelet count were all within the normal limits. A diagnosis of Type 1 von Willebrand Disease (vWD) was established, accompanied by the following prophylactic recommendations:

- Avoid the use of NSAIDs and ASA (acetylsalicylic acid); paracetamol and steroids are permissible.
- In the event of a high-bleeding-risk surgical procedure, the intravenous administration of a plasma-derived product containing FVIII and vWF (e.g., Hemate P^®^ or similar) should be performed at a dosage of 20–100 U/kg every 24 h.
- For minor bleeding events, oral tranexamic acid at a dosage of two 500 mg vials should be taken three times daily for 3–6 days.

Additionally, it is advised to repeat the aforementioned tests for family members suspected of having a bleeding diathesis.

**Figure 11:**
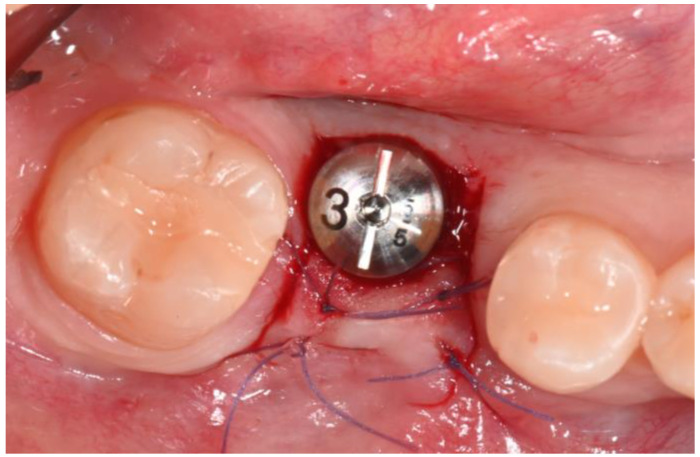
Second-stage surgery: roll flap.

After six months, the second-stage surgery was performed to expose the implant. The roll flap technique was employed to increase the amount of keratinized tissue and to manage the bone defect, as it is indicated for improving soft tissue stability around implants and enhancing the gingival architecture. A healing abutment was placed, and the site was sutured with 5-0 Vicryl (Figure 11).

Postoperative care followed the hematologist’s guidelines for minor surgeries, and no bleeding occurred. One week later, the sutures were removed, and a silicone impression was taken for the provisional crown.

**Figure 12:**
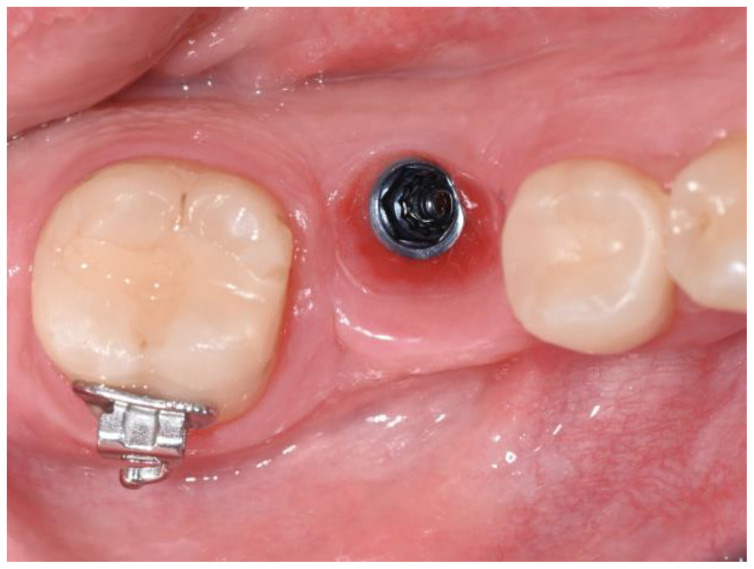
Tissue conditioning.

**Figure 13:**
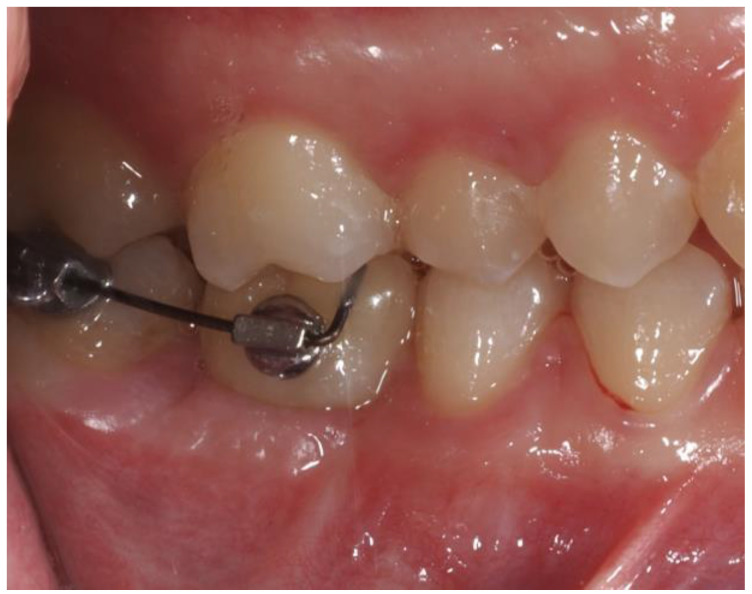
Porcelain-fused-to-metal crown placed over implant.

Two months after tissue conditioning (Figure 12), a porcelain-fused-to-metal crown was placed over the implant (Figure 13. The implant in the 4.6 region was then used as absolute anchorage for upright tooth 4.7, using two tubes on the molars and a 17 × 25 TMA sectional wire, improving the area below the contact point and facilitating proper cleaning of the site (Figure 13).

## 3. Discussion

Despite Von Willebrand diseases (vWD) being the most common inherited bleeding disorder, they often present diagnostic challenges. Patients with vWD type 1 typically exhibit mild and sporadic symptoms, with hematological coagulation parameters frequently appearing normal. This case report details a female patient with an undiagnosed bleeding disorder who underwent implant surgery, resulting in significant postoperative bleeding and a subsequent diagnosis of vWD.

It is not unusual for previously undiagnosed individuals to experience their first excessive bleeding episode during oral or dental procedures, even those typically considered minor, such as endodontic treatment [18]. To date, there are no documented case reports concerning implant placement or orthodontic mini-screws that have led to an incidental diagnosis of vWD.

The signs and symptoms of Type 1 vWD often manifest sporadically throughout the patient’s life, with coagulation screening tests yielding normal results [19]. While a global test like bleeding time might show prolonged results, its limited reproducibility generally excludes it from standard preoperative laboratory panels [20]. In this case, the singular bleeding episode did not recur during other surgical occasions, thus not warranting immediate suspicion of a bleeding diathesis. The hemorrhagic event occurred during a relatively invasive procedure, which had no prior precedent in the patient’s clinical history, as she had never undergone any surgical intervention before.

Furthermore, the patient belongs to the blood group O positive, which is associated with lower-than-normal levels of von Willebrand factor (vWF) [21]. In fact, the ABO blood group’s influence on VWD is relevant, as individuals with blood type O generally exhibit lower baseline levels of von Willebrand factor (VWF) compared to those with types A, B, or AB. This variation is believed to be due to genetic differences influencing VWF clearance rates. Specifically, type O individuals tend to have a shorter half-life of circulating VWF, potentially increasing the severity of bleeding symptoms in those with underlying VWD. In this case, the patient’s blood group, type O, may have contributed to her lower VWF levels and thus exacerbated the bleeding episode observed postoperatively [21,22].

It remains to be determined whether any triggering factors contributed to the bleeding episode, particularly in relation to medications administered before and after the procedure. The literature indicates that NSAIDs, particularly acetylsalicylic acid (ASA), can significantly affect platelet function; however, evidence regarding ibuprofen’s impact in this context is limited [22].

In clinical practice, dentists should exercise caution when screening for blood disorders through detailed anamnesis and clinical evaluation. Many guidelines advise against indiscriminate coagulation screening tests prior to surgery or other invasive procedures to predict postoperative bleeding in unselected patients [22]. A thorough preoperative inquiry into medication history and previous bleeding symptoms may be more predictive of bleeding risk after tooth extraction.

Abnormal gingival bleeding, a common oral condition, may indicate coagulation dysfunction, such as hemophilia, platelet deficiency, or vWD, and may also be influenced by the use of antithrombotic drugs [23]. However, gingival bleeding can also be a primary symptom of plaque-induced gingivitis and untreated periodontal disease, particularly in vWD patients, where gingival inflammation may trigger bleeding rather than indicate an underlying bleeding disorder [23].

While a poorly structured bleeding history does not predict postoperative bleeding, research indicates that the predictive power of such history is contingent on the precise questions asked. A structured interview can be an effective screening tool, even though unstructured bleeding histories have been found to have limited predictive value for postoperative complications. In this context, the ISTH Bleeding Assessment Tool (ISTH-BAT) may be beneficial. This tool comprises 14 categories assessing bleeding symptoms retrospectively, and a high bleeding score is associated with the presence of an inherited bleeding disorder [24]. However, it is important to note that some studies suggest that the ISTH-BAT does not reliably identify patients at increased risk of future bleeding events [23].

In this case report, the ISTH-BAT, administered after the excessive bleeding event, yielded a score of 6, indicating a significant risk of further bleeding. The patient also presented with cutaneous bruising. However, it is important to note that the ISTH-BAT is not specific for diagnosing vWD, and further specialized examinations were necessary.

## 4. Conclusions

To prevent the risk of excessive bleeding, before surgery, all patients should be screened through precise questions on bleeding history. The ISTH-BAT could be an effective predictor for identifying patients who may be at risk. If an abnormal score is obtained, further specific hematological tests should be conducted.

## Data Availability

The data are available on reasonable request from the corresponding authors.
